# Inhibition of Exosome Release Sensitizes U937 Cells to PEGylated Liposomal Doxorubicin

**DOI:** 10.3389/fimmu.2021.692654

**Published:** 2021-06-04

**Authors:** Shirin Hekmatirad, Milad Moloudizargari, Ali Akbar Moghadamnia, Sohrab Kazemi, Mousa Mohammadnia-Afrouzi, Maryam Baeeri, Fatemeh Moradkhani, Mohammad Hossein Asghari

**Affiliations:** ^1^ Student Research Committee, Babol University of Medical Sciences, Babol, Iran; ^2^ Department of Immunology, School of Medicine, Shahid Beheshti University of Medical Sciences, Tehran, Iran; ^3^ Department of Pharmacology and Toxicology, School of Medicine, Babol University of Medical Sciences, Babol, Iran; ^4^ Cellular and Molecular Biology Research Center, Health Research Institute, Babol University of Medical Sciences, Babol, Iran; ^5^ Department of Immunology, School of Medicine, Babol University of Medical Sciences, Babol, Iran; ^6^ Toxicology and Diseases Group, Pharmaceutical Sciences Research Center (PSRC), The Institute of Pharmaceutical Sciences (TIPS), Tehran, Iran; ^7^ Department of Toxicology and Pharmacology, Faculty of Pharmacy, Tehran University of Medical Sciences (TUMS), Tehran, Iran; ^8^ Department of Medicinal Chemistry, Faculty of Pharmacy and Pharmaceutical Sciences Research Center, Tehran University of Medical Sciences, Tehran, Iran

**Keywords:** extracellular vesicles (EVs), exosome, cancer, drug resistance, liposomal doxorubicin, virtual screening

## Abstract

**Aims:**

Acute myeloblastic leukemia (AML) is the most common type of acute leukemia in adults. Despite numerous treatment strategies including chemotherapy and radiotherapy, a large number of patients do not respond to treatment and experience relapse. The main problem of these patients is the development of resistance to anti-cancer drugs. Therefore, any endeavor to reduce drug resistance in these patients is of high priority. In general, several mechanisms such as changes in drug metabolic pathways, drug inactivation, drug target alterations and reduced drug accumulation in the cells contribute to drug resistance of cancer cells. In this context, evidence suggests that exosomes could reduce drug resistance by removing drugs from their parent cells. In the present study, we aimed to investigate the effects of exosome release inhibition on the resistance of U937 cells to PEGylated liposomal doxorubicin (PLD).

**Main Methods:**

In order to find a suitable ABCG2 (ATP-binding cassette sub-family G member 2) transporter substrate, virtual screening was performed among a list of drugs used in leukemia and PLD was selected. U937 cells were treated with PLD with/without co-treatment with the exosome release inhibitor, GW4869. Released exosomes within different study groups were isolated and characterized to determine the differences between groups. Doxorubicin presence in the isolated exosomes was also measured by high performance liquid chromatography (HPLC) to confirm drug export through the exosomes. Finally, the effect of exosome inhibition on the cytotoxicity of PLD on U937 cells was determined using different cytotoxicity assays including the standard lactate dehydrogenase (LDH) release assay and the flow cytometric analysis of apoptotic and non-apoptotic cell death.

**Key Findings:**

GW4869 treatment caused a significant decrease in the exosome release of U937 cells compared to the untreated cells, as evidenced by the reduction of the protein content of the isolated exosomes (P<0.05). Co-treatment with GW4869 significantly increased cytotoxic cell death in the groups treated with 0.5 and 1 µM PLD, compared to the same groups without GW4869 co-treatment (P<0.05). Interestingly, co-treatment with GW4896 and 0.5 µM PLD was enough to induce the same cytotoxic effect as that of the sole 1 µM PLD group.

**Significance:**

Our findings showed that U937 cells increase their resistance against the cytotoxic effects of PLD through the exosome-mediated expelling of the drug. Inhibition of exosome release could prevent PLD efflux and consequently increase the vulnerability of the U937 cells to the cytotoxic effects of PLD. Our results along with prior studies indicate that the integration of exosome release inhibitors into the common PLD-containing chemotherapy regimens could significantly lower the required concentrations of the drug and consequently reduce its associated side effects. Further studies are warranted to identify clinically safe inhibitors and investigate their clinical efficacy.

## Introduction

Leukemias are a group of hematological malignancies in which mutated hematopoietic progenitors produce great number of abnormal leukocytes, called blasts, which accumulate in bone marrow, tissues and blood. Based on prognosis, myelogenous leukemia is classified as acute myelogenous leukemia (AML) and chronic myelogenous leukemia (CML) (1).

AML is mainly a disease of the elderly and its prevalence is higher in people over 65 years of age. An estimated 21,450 cases were diagnosed in 2019 in the United States, and more than 10,900 patients died this year, accounting for 62% of leukemia-associated deaths. Since, AML’s five-year survival rate is less than 10%, it is very difficult to manage and treat patients (2, 3). Despite advances in the treatment of AML and the availability of a variety of treatment options from chemotherapy and targeted therapies to stem cell transplantation, a large number of patients experience recurrence, which is mainly caused by drug resistance. Accordingly, new strategies are needed to maximize response to initial treatment and increase survival period (4).

Cancer cells employ various mechanisms, collectively known as drug resistance mechanisms, to escape the destructive effects of therapeutic agents including chemotherapy drugs and all other types of anti-cancer agents (5). Some types of cancer cells are inherently resistant to treatment and, therefore immediately demonstrate resistance therapy, while others initially respond to treatment and gradually develop acquired resistance following subsequent exposure (6). Although induction chemotherapy with cytarabine with anthracyclines results in an acceptable response in about 70% of AML patients, unfortunately, recurrence occurs following acquired drug resistance in these patients (7).

Exosomes, a widely studied subset of extracellular vesicles, are nanovesicles with a lipid bilayer membrane. These nanovesicles are of endosomal origin and carry different compositions of lipids, proteins and nucleic acids depending on the cell from which they originate as well as the conditions in which they are produced and secreted (8, 9). Through the transportation of such content between different cells, exosomes facilitate intercellular communication and therefore, play critical roles in various physiological and pathological processes in the body. Cancer cells exploit this ability of exosomes to change their microenvironment in favor of tumor spread (10–12). A great deal of evidence suggests that exosomes can induce resistance of cancer cells to drugs due to their ability to pack and transport biological cargo including the therapeutic agents and other molecules responsible for drug resistance such as certain microRNAs, drug transporters, etc. (13, 14).

The presence of drug transporters in the exosomes is closely related to the concept of drug resistance mediated by these vesicles. These transporters have been shown to pass through exosomes to reach their target cells and eventually induce their drug resistance. Furthermore, through their presence within the membrane of exosomes, these transporters may facilitate the entry of the drugs into exosomes and the subsequent exosome-mediated expulsion of the drugs from the cells of origin (15, 16). Studies have shown that drugs such as topotecan and riboflavin are loaded into ABCG2-rich (ATP-binding cassette sub-family G member 2) exosomes and thereby, removed from the cells. Therefore, the possible role of this transporter in the exosome-mediated efflux of these drugs was highlighted (17). It has been suggested that these transporters are located in the reverse direction on the membrane of the exosomes, thus endowing the drugs a free passage into these vesicles (16). Therefore, the use of exosome inhibitors could be a new strategy to increase the sensitivity of cancer cells to treatment. In the recent years, various attempts on the pharmacological inhibition of exosome release have been made and the effectiveness of such inhibitors such as neticonazole, ketotifen, cannabidiol, and GW4869 has been demonstrated (18, 19).

Exosomes secreted from AML cell lines as well as primary AML blasts have the ability to regulate tumor microenvironment and cell activity in favor of cancer progression and induction of drug resistance (20). Szczepanski et al. have shown that AML cells are able to secrete larger amounts of exosomes compared to healthy controls and have also shown that the molecular profiles of their exosomes are different (21). U937 is an AML cell line from which secreted exosomes have been extracted and studied in various studies. Exosomes secreted by U937 cells have also been used in studies on the drug resistance of AML to chemotherapeutic agents (22).

Doxorubicin is an anthracycline, widely used in the treatment of various malignancies. Despite the fact that this drug is one of the most widely-utilized chemotherapeutic drugs, due to its serious side effects especially the cardiac toxicity of its cumulative doses, its use has been limited in recent years (23). Nanotechnology has relatively helped solve this problem by encapsulating doxorubicin in liposomes to increase its efficiency and reduce its toxicity. PLD is a liposomal formulation in which doxorubicin is loaded into liposomes with methoxypolyethylene glycol on their surface. However, there is still a strong need for the development of novel strategies to cope with this limitation of doxorubicin (24).

In the present work, we made an attempt to examine the possible drug-sensitizing effects of exosome inhibition in combination with the treatment of AML cells with a cytotoxic agent. Given the previously demonstrated role of ABCG2 transporters in exosome-mediated expulsion of drugs from cancer cells, after a bioinformatics screening of anti-leukemic drugs, doxorubicin was found to be a desirable ABCG2 substrate. Based on the limitations of doxorubicin due to its side effects the liposomal formulation, PLD, was selected to be used for the treatments. GW4869, a commonly used exosome inhibitor, was used to investigate the effectiveness of such a strategy in increasing the sensitivity of U937 cells to doxorubicin.

## Materials and Methods

### Reagents and Antibodies

PEGylated liposomal doxorubicin was obtained from Janssen (Belgium). Exo-spin™ exosome purification kit was obtained from Cell Guidance systems (UK). CyQUANT™ LDH cytotoxicity assay kit and 3.9-μm latex beads were purchased from Invitrogen (USA). Exosome-free fetal bovine serum (FBS) was from System Biosciences (USA) and RPMI 1640 was from Capricorn (Germany). BCA protein assay kit was purchased from Parstous Biotechnology (Iran). Anti-CD63/PE antibody and Isotype control mouse IgG/PE was from BioLegend (USA). Annexin V-FITC/PI kit was obtained from MabTag (Germany).

### Virtual Screening

The crystal structure of ABCG2 (PDB: 6VXI) was obtained from Protein Data Bank (http://www.rcsb.org/). After the removal of the co-crystallized ligand (mitoxantrone), several preparations were made to the receptor *via* merging the non-polar hydrogens and assigning Kollman charges. The structure data file (SDF) formats of 46 anti-leukemia chemotherapeutic agents were obtained from PubChem (https://pubchem.ncbi.nlm.nih.gov). Using the PyRx software, pdbqt conversion and energy minimization was performed. PyRx was also used to perform the virtual screening. The grid box was calculated based on the co-crystallized ligand. The best ligand conformation was attained based on the lowest docking energy. Discovery Studio visualizer v16.1.0 and PyMol 2.3.6 were used to study the ligand-receptor interactions.

### Cell Culture Treatment

U937 cell line was obtained from the Pasteur Institute of Iran. The cells were grown in RPMI-1640 supplemented with 10% fetal bovine serum (FBS) and 1% penicillin and streptomycin at 5% CO_2_ and a temperature of 37°C. U937 cells at their log phase of growth were first washed with cold phosphate-buffered saline (PBS) and then seeded in T-75 flasks containing complete medium supplemented with exosome-depleted FBS. The cells were allocated to a control group, which remained untreated and seven study groups including a group solely receiving 20 µM GW4869, and six other groups treated with three different concentrations of PLD (0.5, 1 and 2 µM) with or without 20 µM GW4869. The incubation time for all the treatments was 24 h.

### Exosome Isolation

In order to confirm exosome production and release by U937 cells, exosomes were isolated from the cells in different groups and characterized. At the end of the incubation time, the cells were centrifuged at 16000 g for 20 min to precipitate the cells. The supernatants were then collected and centrifuged once again to eliminate any remaining cellular debris and filtered through a 0.2 µM Nanopore filter. The filtered supernatants were subjected to the steps indicated in the Exo-spin commercial exosome isolation kit. Briefly, the buffer A supplemented within the kit, was added to the supernatants and incubated at 4°C overnight. Then, the samples were centrifuged at 16000 g for one hour and the supernatants were passed through the isolation columns. Finally, the isolated exosomes in each group were obtained at a total volume of 200 µL. The purified exosomes were stored at -80°C until further analysis.

### Exosome Characterization and Quantification

#### Transmission Electron Microscopy

Size and morphology analysis of the isolated exosomes was performed by transmission electron microscopy (TEM) based on the method described by Moloudizargari et al. (8).

#### BCA Protein Assay

Parstous BCA protein assay kit was used to quantify the protein content of the purified exosomes. To do this, a standard curve was obtained using 9 serial dilutions of BSA and the absorbance of each sample was converted to µg/mL protein using this curve.

#### Flow Cytometry

The purified exosomes were loaded onto latex microbeads to render them suitable for flow cytometry studies. Accordingly, 5 μg of the isolated exosomes was mixed with 10 μL of latex microbeads. After 15 minutes of incubation at room temperature, PBS was added to the exosome and microbead mixture to reach a volume of 1 ml and was incubated overnight. Afterwards, 100 mM glycine was used to block the remaining unspecific binding sites on the microbeads. Following 30 minutes of incubation at room temperature, the resulting mixture was centrifuged for 4 minutes at 4000 g and the pellet was washed three times with PBS containing 0.5% BSA. Subsequently, latex-loaded exosomes were stained with PE-conjugated anti-CD63 antibody and the corresponding isotype control and examined by flow cytometry. Exosome-free microbeads stained with the same antibody were also used as a control for nonspecific antibody binding.

### HPLC

The presence of doxorubicin in exosomes secreted from U937 cells was evaluated by HPLC. To do this, the intact structure of the isolated exosomes was sequestrated using a lysis buffer containing triton X-100 and doxorubicin concentration was subsequently measured in the solution. In summary, the mobile phase consisted of acetonitrile and water (PH = 3) with a ratio of 70:30 and the flow rate of the mobile phase was 1 mL/minute. The absorption spectrum at 233 nm was investigated by a UV detector. To draw a standard curve, four solutions containing serial dilutions of doxorubicin (5.2, 5, 10 and 20 ppm) were prepared and injected into the device.

### Viability Assays

In order to quantify the extent of cytotoxic cell death in the studied groups and to investigate the effect of exosome inhibition on PLD cytotoxic function, flow cytometric analysis of cell death was done following annexin V/PI staining and the standard lactate dehydrogenase (LDH) release assay was also performed.

#### Annexin V/PI Staining

To study cell death by flow cytometry, U937 cells were first seeded in 6-well plates and incubated with different concentrations of PLD for 24 hours at 37°C and 5% CO2. After incubation, the cells were collected and washed with PBS and stained with annexin V/PI according to the protocol provided within the Mabtag kit. Finally, the data was acquired by a FACS Calibur flow cytometer and analyzed by the FlowJo software version X.

#### LDH

This assay is based on the measurement of the LDH enzyme released from damaged cells. Invitrogen commercial kit was used to perform the experiments. U937 cells were seeded in a 96-well plate and treated with different concentrations of PLD. At the end of the incubation time, 50 μL of each well was transferred to a new 96-well plate and 50 μL of the reaction mixture, provided in the kit, was added to each well and mixed gently. After 30 minutes of incubation, 50 μL of the stop solution was added to each well and after one hour the absorption was measured at 490 nm and 680 nm. The percentages of specific cytotoxicity of PLD in different study groups were then calculated using the following formula, where the obtained ODs were directly used to perform the calculations:

$$\%\;Cytotoxicity=\;\left[\frac{Compound-treated\;LDH\;activity-Spontaneous\;LDH\;activity}{Maximum\;LDH\;activity-Spontaneous\;LDH\;activity}\right]\times 100$$

### Statistical Analysis

The GraphPad Prism 6 software was used to perform all the statistical analysis of the present study. One-way analysis of variances (ANOVA) followed by a post hoc Tukey test was used to determine significant differences between groups and the results were reported as Mean ± SD. All experiments were performed in triplicate. P-value less than 0.05 were considered statistically significant.

## Results

### Virtual Screening and Molecular Docking

**Figure 1:**
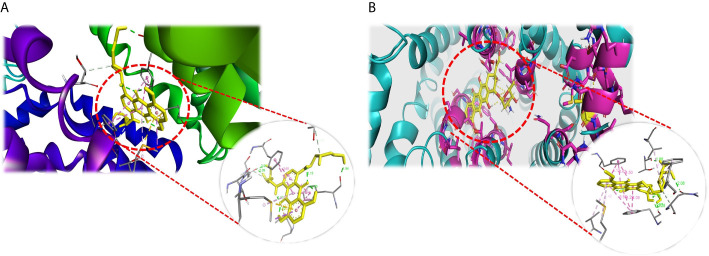
The representation of mitoxantrone **(A)** and doxorubicin **(B)** docked with ABCG2. The ligands (mitoxantrone and doxorubicin) are shown in yellow. The active site residues are displayed as stick and the backbone of the receptor as ribbon.

**Figure 2:**
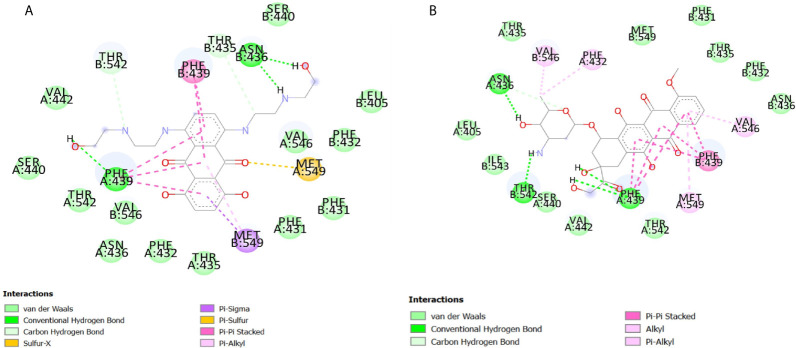
Two-dimensional (2D) representation of the interactions of mitoxantrone **(A)** and doxorubicin **(B)** with ABCG2, generated by discovery studio visualizer v16.1.0. The different residues are marked by colored circles and three-letter abbreviations, and the colors indicate the type of interaction.

In order to find the best substrate for the ABCG2 transporter among the screened chemotherapeutic drugs used in the treatment of leukemia, structure-based docking was performed. Doxorubicin had the lowest binding energy among the drugs used in AML treatment (**Table 1**). To evaluate the validity of the screening and the final selected compound, molecular docking by ADT was performed for doxorubicin and mitoxantrone separately. The results of docking showed that doxorubicin had lower binding energy than mitoxantrone and therefore was selected for the present study. Considering the superiority of the liposomal form of this drug over its standard form, PLD was finally selected to be used in the experiments. The interaction of these two ligands is demonstrated in **Figures 1** and **2**.

**Table 1:** Top 10 compounds obtained in virtual screening.

	Compound	Binding affinity	RMSD
1	Nilotinib	-12.8	0
2	Ponatinib	-12.2	0
3	Imatinib	-11.8	0
4	Ibrutinib	-11.6	0
5	Acalabrutinib	-11.3	0
6	Duvelisib	-11	0
7	Epirubicin	-10.4	0
8	Doxorubicin	-10.3	0
9	Idarubicin	-10.3	0
10	Enasidenib	-10.3	0

RMSD, Root-mean-square deviation. The binding energies and RMSDs of these compounds are given in this table. Among these compounds, doxorubicin was with the lowest binding energy and RMSD, which is the first common compound in the treatment of AML in this table.

### Exosome Characterization

**Figure 3:**
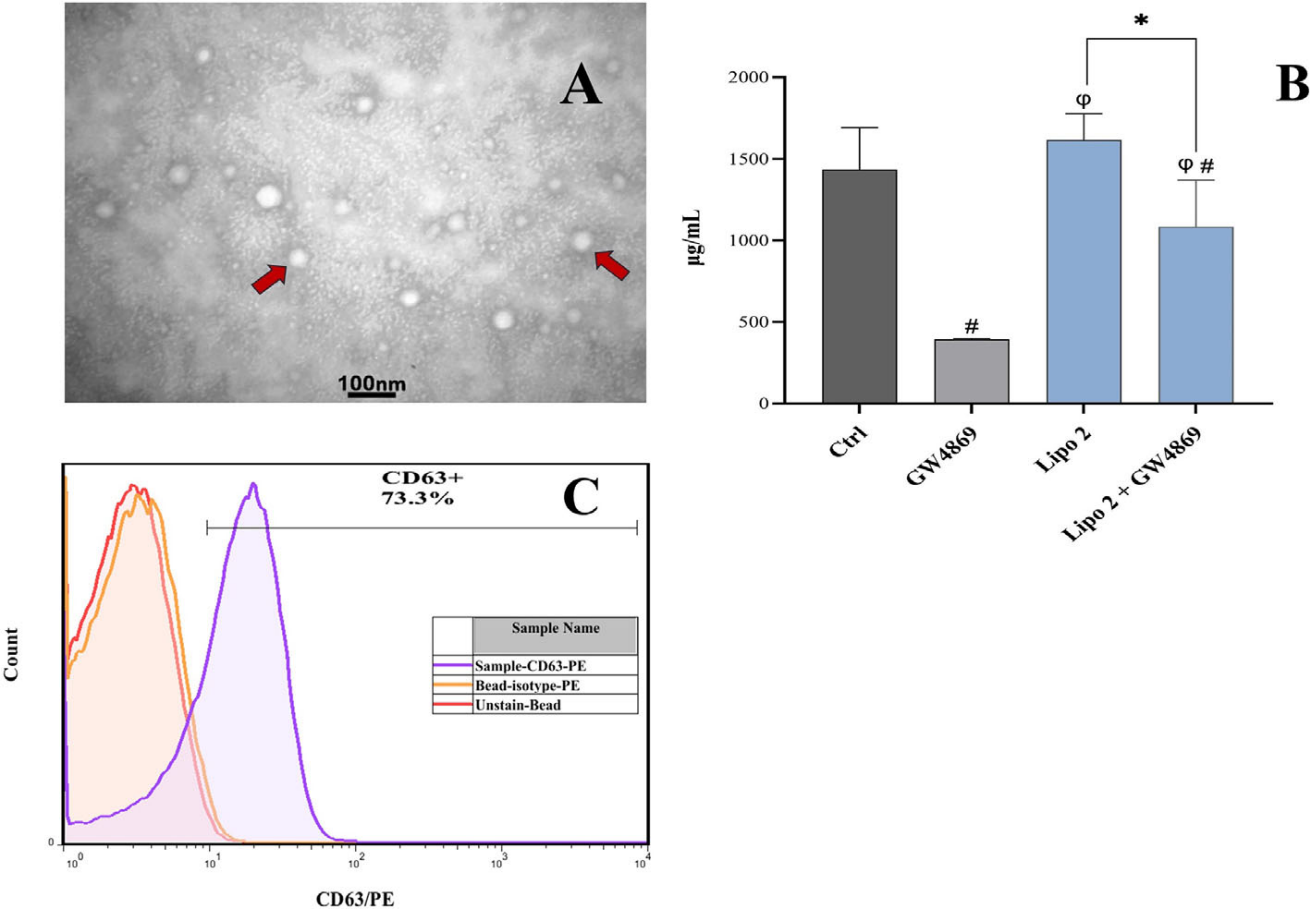
Characterization of exosomes isolated from U937 cells. **(A)** TEM micrograph shows cup-shaped morphology, **(B)** BCA protein concentration assay reveals decreased protein concentration after GW4869 treatment, ^#^Significantly different compared to the control (P < 0.05), *Significantly different compared to the same concentration with/without GW4869 (P < 0.05), ^φ^Significantly different compared to the GW4869-treated group, **(C)** The isolated U937 exosomes are positive for the CD63 marker.

Exosomes extracted and purified from U937 cells in different study groups were collected. The micrographs of morphological evaluation by TEM displayed round, cup-shaped nano-vesicles with a size range of 30-150 nm (**Figure 3A**). Exosomal protein concentration was measured as an indicator of the extent of exosome release among different study groups. The exosomal protein concentration of the sole GW4869 group and the combinational 2 μM PLD + GW4869 group were significantly different from that of the control group; however, sole treatment with 2 μM PLD did not induce any significant change in the exosomal protein concentration of the cells (**Figure 3B**). Although exosomes are originated and secreted from different cells, their production process is the same in all cells; therefore, a number of markers can be used to identify the population of exosomes. For this purpose, we measured the presence of the tetraspin CD63 on the surface of the exosomes by flow cytometry. We also used an isotype control to ensure the specificity of the results. The results confirmed the expression of CD63 in the prepared samples of exosomes (**Figure 3C**).

### Confirmation of Doxorubicin Loading in Exosomes

**Figure 4:**
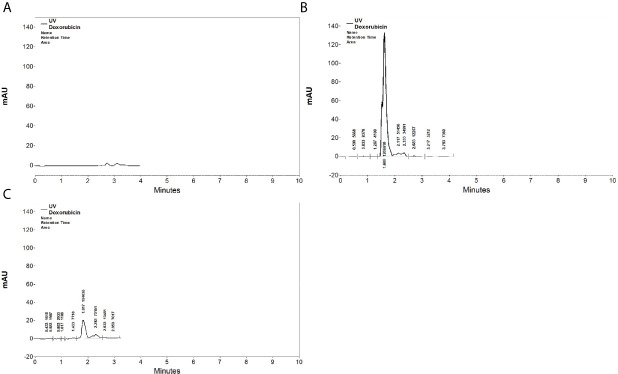
Doxorubicin detection in exosome samples by high-performance liquid chromatography (HPLC). **(A)** Absorption spectrum of digested exosomes extracted from untreated U937 cells, **(B)** Absorption spectrum of 10 ppm doxorubicin standard sample, **(C)** Absorption spectrum of digested exosomes extracted from U937 cells treated with 2 μM PLD. The presence of doxorubicin in the exosomes and thus the ability of the exosomes to package and export the drug is indicated based on the absorption spectrum of the injected digested exosome sample.

U937 extracted exosomes were examined by HPLC to investigate the loading of doxorubicin into the isolated exosomes. The results showed that doxorubicin can be exported from the U937 cells through exosomes (**Figure 4**).

### Exosome Release Inhibition Is Associated With Increased Cytotoxicity of PLD

In order to investigate the effect of exosome release inhibition on the cytotoxicity of PLD, after the treatment of U937 cells in different study groups, the percentage of cell death was determined with flow cytometry following Annexin V/PI staining and specific cytotoxicity was also calculated using the LDH release assay.

**Figure 5:**
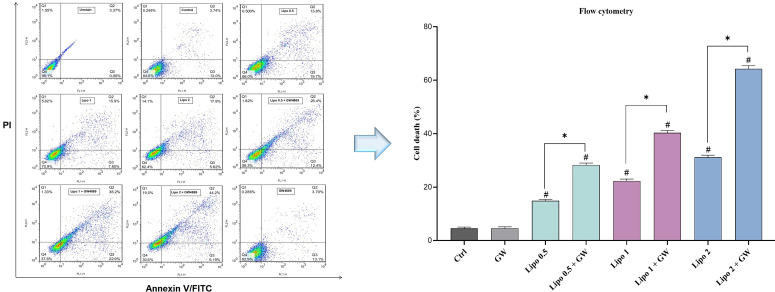
Flow cytometry analysis of cell death in PLD-treated U937 cells in different groups. The graphs represent cell populations undergone complete cell death, which were identified as the sum of PI single positive plus annexin V/PI double-positive populations in each group. Based on the results, co-treatment of U937 cells with GW4869 in addition to different concentrations of PLD could increase its cytotoxicity compared to the corresponding concentrations without GW4869. Sole GW4869 treatment did not have any significant effects on the viability of U937 cells compared to the untreated control (P > 0.05). Ctrl, untreated control; GW, GW4869-treated; Lipo 0.5, 0.5 µM PLD; Lipo 0.5 + GW, 0.5 µM PLD with GW4869; Lipo 1, 1 µM PLD; Lipo 1 + GW, 1 µM PLD with GW4869; Lipo 2, 2 µM PLD; Lipo 2 + GW, 2 µM PLD with GW4869. ^#^Significantly different compared to the control group (P < 0.05), *significantly different compared to the same concentration with/without GW4869 (P < 0.05).

**Figure 6:**
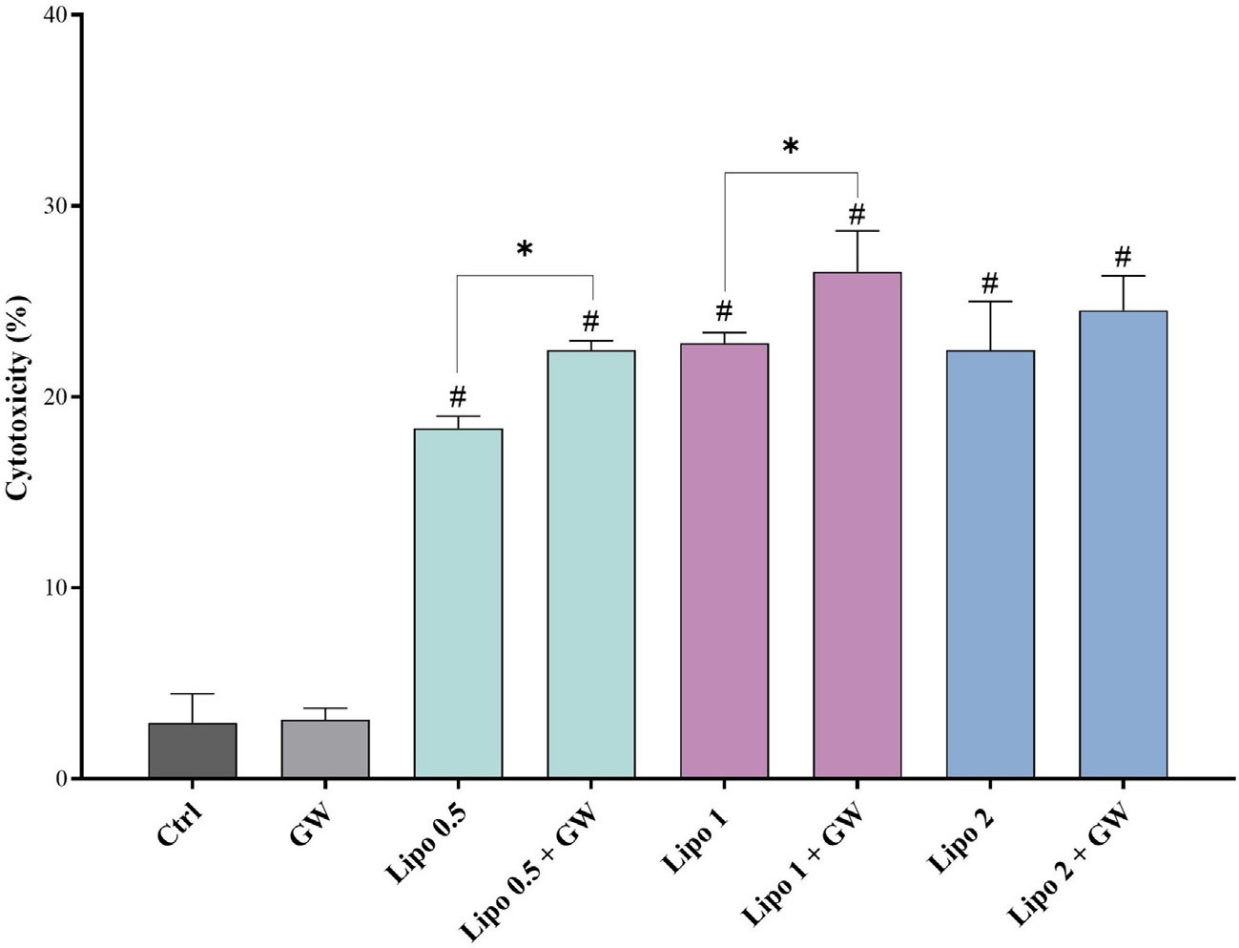
Quantitative analysis of specific cytotoxicity in U937 cells exposed to different concentrations of PLD with/without GW4869 by the LDH release assay. Co-treatment of U937 cells with GW4869 in addition to 0.5 and 1 µM concentrations of PLD could increase its cytotoxicity compared to the corresponding concentrations without GW4869. The combined use of the GW4869 and PLD was able to reduce the required concentration of the drug by half to yield the same cytotoxic effect. Sole GW4869 treatment did not have any significant effects on the specific cytotoxicity of U937 cells compared to the untreated control (P > 0.05). Ctrl, untreated control; GW, GW4869-treated; Lipo 0.5, 0.5 µM PLD; Lipo 0.5 + GW, 0.5 µM PLD with GW4869; Lipo 1, 1 µM PLD; Lipo 1 + GW, 1 µM PLD with GW4869; Lipo 2, 2 µM PLD; Lipo 2 + GW, 2 µM PLD with GW4869. ^#^Significantly different compared to the control group (P < 0.05), *significantly different compared to the same concentration with/without GW4869 (P < 0.05).

The results of flow cytometry indicated that co-treatment of U937 cells with GW4869 in combination with all concentrations of PLD was able to significantly increase its cytotoxicity compared to the same concentrations without GW4869 co-treatment (P<0.05) (**Figure 5**). The results of the LDH assay were almost in line with flow cytometry indicating a significant difference between the specific cytotoxicity calculated for 0.5 and 1 µM concentrations of PLD compared to the corresponding concentrations with GW4869 co-treatment (P<0.05) (**Figure 6**).

## Discussion

AML is a life-threatening blood malignancy that despite the advances in its therapy, more than 85% of the patients fail to respond to treatment. Previous studies have suggested that drug resistance is the key responsible factor for the treatment failures and short-term survival in these patients (4).

Cellular communication between cancer cells and their host cells forms a complex network that affects the progression potential of cancer cells. Classically, this network is defined by the cellular secretory molecules or the direct interaction of cells with each other. However, in the recent years, another fundamental mechanism of inter-cellular communication has been proposed based on the release of extracellular vesicles. Over the years, the formation, function and packaging process of extracellular vesicles, especially exosomes, have received much attention. Numerous studies have shown the roles of exosomes in various diseases including autoimmunity, neurodegenerative diseases, inflammation and cancer (15). Exosomes also play a key role in drug resistance induction and have been described as its important mediators. In this regard, studies have shown that exosomes cause drug resistance by exporting chemotherapeutic agents from cells. Tumor acidity increases the secretion of exosomes by the cells and drugs can be trapped within the acidic exosomes released by these cells (25, 26). This phenomenon of sequestration of drugs by the tumor-derived exosomes reduces the concentration of the drug in the tumor cells and is considered as a mechanism of drug resistance in the cell(s) of origin (27, 28).

Drug resistance is generally associated with multidrug resistance proteins (MDR). These proteins belong to the ABC transporter family, which carry various molecules along the plasma membrane. These transporters are involved in inducing exosome-mediated drug resistance, especially in the exosome-releasing cells (29, 30). ABCG2 is one of the key transporters and studies on the ABCG2-rich exosomes have shown that drugs such as riboflavin, topotecan and methotrexate can be expelled from the cells through exosomes (31). It has been suggested that the PI3K/Akt pathway may play a role in the ABCG2 arrangement on the exosome membrane (28). Accordingly, in this study, we screened a library of anti-leukemia drugs to find the best ABCG2 transporter substrate among different chemotherapeutic agents employed in the treatment of leukemia. During this screening, doxorubicin had the highest score among the drugs used for AML. In the next step, we performed in vitro studies to investigate the potential of AML cells to pack doxorubicin into exosomes and expel it from the cell(s) of origin. Furthermore, we studied the impact of exosome inhibition on the cytotoxic effect of PLD on U937 cells.

Doxorubicin, also known as Adriamycin, is the most widely used anthracycline, which has shown significant therapeutic effects in many types of cancers and is one of the most potent chemotherapy drugs. However, its application is limited due to its toxicity profile, especially cardiac toxicity (32).

Unfortunately, like all other cytotoxic agents, doxorubicin does not specifically target tumor markers and has the potential to affect the growth and function of host cells. The severity of side effects depends on the prescribed dose. In addition, doxorubicin has a very short half-life with a wide non-selective tissue distribution. Therefore, like many other anticancer drugs, effective treatment with doxorubicin often requires high concentrations, which can exacerbate toxic side effects due to the lack of selectivity (33).

Nanotechnology methods have proven promising to overcome the various limitations of cancer treatment. The high surface-area-to-volume ratio and high surface ligand density are among the important characteristics of nanoparticles employed herein. Nanoparticles increase the local concentration of the drug by controlled transport and release of the drug. PLD is among the organic nanoparticles of doxorubicin, which are superior to its classic form in terms of efficacy and less serious side effects (34).

In the present study, GW4869, a common exosome inhibitor, was used to investigate the role of exosomes in the induction of drug resistance to PLD. The inhibitory effect of GW4869 on exosome release has been investigated in several in vitro and in vivo studies. In one of these studies, Nakamura et al. showed that GW4869, by inhibiting the release of ovarian cancer exosomes, reduced the cancer invasion. It reduced the exosome release without inducing toxicity or affecting cell viability. The results of this study showed that targeting the ceramide pathway and consequently exosome release could be a good option to make the current ovarian cancer treatments more effective (35). In another study, Cai et al. examined the effect of bone marrow-derived mesenchymal stem cells (BMSCs)-derived exosomal miR-9-3p on bladder cancer cells. BMSCs and cancer cells co-culture showed decreased survival and invasion of cancer cells and increased apoptosis. GW4869 was used to investigate the role of exosomes and found that inhibition of exosomes increased the survival and invasion of cancer cells and decreased apoptosis. According to their observations, GW4869 alone had no effect on cell viability (36). In another study, Richards et al. found that GW4869 inhibited exosome secretion from gemcitabine-treated cancer-associated fibroblasts and also reduced drug resistance in pancreatic cancer. Considering the important role that they observed for GW4869 in reducing drug resistance, it was concluded that the use of GW4869 and exosomal inhibitors in general along with chemotherapy drugs can be an effective treatment strategy (37). In our study, after extracting the exosomes, we evaluated the CD63 surface marker, protein content and morphology to confirm the exosome produced by U937 cells.

Studies have shown that compared to the healthy individuals, the plasma of AML patients contains higher levels of exosomal protein. These exosomes have also been shown to be different in terms of their molecular profile among AML patients and healthy individuals. Moreover, exosomal protein levels in AML patients appear to reflect the extent of the disease and are associated with recurrence after treatment. Regarding treatment-related changes in exosomal protein levels, it appears that its significant reduction after chemotherapy is associated with a decrease in AML blasts in the bone marrow, resulting in lower exosome secretion (38). In the present study, the protein concentration in the GW4869-treated group was significantly lower than the control group, which indicates the successful inhibition of exosome release by this agent. GW4869 in combination with 2 μM PLD also significantly reduced the protein concentration and thus inhibited the release of exosomes. Various studies have shown that chemotherapy drugs can increase the secretion of exosomes from cancer cells (39). Increased exosome secretion of hepatocarcinoma cells induced by paclitaxel and carboplatin (40) and the stimulation of exosome release by doxorubicin in Balb/C mice (41) are only some of many examples supporting this notion. A possible mechanism for such an increase could be the effect of chemotherapy drugs on the induction of ceramide synthesis (42). Studies have shown that doxorubicin promotes the nSMase2 enzyme function, resulting in the production of ceramide (42). Accordingly, doxorubicin interferes with the function of GW4869 by affecting the ceramide production pathway, which may explain why GW4869 was not as efficient in combination with PLD as its sole use. Importantly however, it was still successful enough in lowering exosome release compared to the sole PLD group, which was the major goal of our study.

After confirming the successful isolation of exosomes and the inhibitory effect of GW4869 on exosome release, the presence of doxorubicin in the exosomes was investigated by HPLC. Finally, we evaluated the effect of this inhibition on drug resistance and cell death. For this purpose, cell death of PLD-treated U937 cells with/without GW4869 was assessed by the LDH assay and flow cytometry. Investigation of cell death in both tests showed that the use of GW4869 with each concentration of PLD increased cell death compared to the sole use of PLD at the same concentrations. Accordingly, it is possible that the inhibition of exosome release also contributed to the accumulation of PLD in the U937 cells and thus increased the sensitivity of these cells to treatment.

Interestingly, the extent of cytotoxic effect in the 1 µM PLD-treated group and the combinational 0.5 µM PLD and GW4869 treated group were not significantly different. This finding indicate that the concomitant use of lower concentrations of PLD with an exosome inhibitor could give rise to a cytotoxic effect similar to higher concentrations of the drug alone.

Increasing drug accumulation in cancer cells is a strategy to improve the effectiveness of chemotherapeutic drugs. Preventing drugs from leaving the cell via inhibiting the release of exosomes is a means of such strategy, which could increase the sensitivity of tumor cells to chemotherapy (43). On the other hand, the required concentration of chemotherapeutic agents may potentially be reduced, using a combination of these agents with exosome inhibitors. This could be a promising strategy in the design of novel treatment protocols by reducing the side effects of chemotherapy and, at the same time, maintaining the beneficial cytotoxic effects (44).

Our results were in accordance with as the results of a study by Koch et al. in which they used indomethacin to inhibit exosome release in B cell lymphoma. Indomethacin down-regulated the ABCA3 transporter, which is involved in the packaging and secretion of drugs such as anthracyclines into the exosomes. By increasing intracellular drug accumulation, indomethacin improved the efficacy of doxorubicin and counteracted drug resistance (45). In another study, chloramidine and bisindolylmaleimide-I were used to inhibit exosome release from PC3 and MCF7 cancer cell lines. Concomitant use of these two inhibitors and Fluorouracil (5-FU) increased the antineoplastic activity of 5-FU, which may be due to the increased drug accumulation in these cells (46). In agreement with the results of the present study, Khan et al. also showed that reducing the release of doxorubicin-containing exosomes from cancer cells by ketotifen, a mast cell stabilizer, could improve response to the drug, which was a confirmation of doxorubicin removal from cancer cells by exosomes (47).

## Conclusion

The findings of this study provide evidence on the capability of AML cells to expel doxorubicin into exosomes leading to their resistance to PLD. Our results are consistent with the results of many studies on the role of exosomes in various diseases and emphasize the importance of exosomes as potential targets for the design of optimal treatment regimens for cancer.

As the number of exosome-focused clinical trials, from proteomic evaluation of secreted exosomes to engineered exosomes as drug delivery vehicles, has been increasing in recent years, there is a lack of clinical trials on drug resistance and evaluation of the clinical efficacy and safety of exosome inhibitors in combination with conventional treatment regimens. Finally, designing clinical trials on the role of exosomes and the effectiveness of their inhibition in managing drug resistance of various cancers to therapy is warranted.

## Data Availability Statement

The raw data supporting the conclusions of this article will be made available by the authors, without undue reservation.

## Author Contributions

SH and MHA postulated the main idea and prepared the main backbone of the paper. FM, MB, and MM performed the in-depth literature search and drafted the paper, SK and MM-A supervised the methods, MHA and AAM made final revisions and supervised the whole work. All authors contributed to the article and approved the submitted version.

## Funding

This work is the outcome of a Master thesis by Shirin Hekmatirad at Babol University of Medical Sciences (Grant No. IR.MUBABOL.HRI.REC.1398.157). 

## Conflict of Interest

The authors declare that the research was conducted in the absence of any commercial or financial relationships that could be construed as a potential conflict of interest.
